# Validating Thermal Lethality to *Salmonella enterica* in Chicken Blood by Simulated Commercial Rendering

**DOI:** 10.3390/microorganisms8122009

**Published:** 2020-12-16

**Authors:** Caleb Wong de la Rosa, Kourtney A. Daniels, Rosana G. Moreira, Chris R. Kerth, Thomas M. Taylor

**Affiliations:** 1 Department of Animal Science, Texas A&M AgriLife Research, College Station, TX 77843-2471, USA; calebwong19@gmail.com (C.W.d.l.R.); c-kerth@tamu.edu (C.R.K.); 2 Department of Food Science and Technology, Texas A&M AgriLife Research, College Station, TX 77843-2253, USA; kdaniel5@tamu.edu; 3 Department of Biological and Agricultural Engineering, Texas A&M AgriLife Research, College Station, TX 77843-2117, USA; rmoreira@tamu.edu

**Keywords:** *Salmonella*, rendering, chicken blood, validation, lethality, blood meal

## Abstract

The U.S. rendering industry produces materials for use in further processed animal foods and feeds and is required to scientifically validate food safety hazard control. This study aimed to provide lethality validation for *Salmonella enterica* during simulated commercial rendering of whole chicken blood. Chicken blood was inoculated with a blend of multiple serovars of the pathogen (*S.* Heidelberg, Typhimurium, Senftenberg) and subjected to heating at 82.2, 87.8, or 93.3 °C; surviving cells were enumerated incrementally up to 5.0 min. Survivor data were modeled using the GInaFiT 1.7 freeware package. *D*-values and t_7*D*_ (time to a 7.0 log_10_-cycle inactivation) values were generated from best-fit model parameters. Predictive modeling analysis revealed that the survival curves of *Salmonella* possessed log-linear components but also possessed shoulder and/or tail components. Mean *D*-values declined from 0.61 to 0.12 min as heating temperature was raised from 82.2 to 93.3 °F, respectively, differing by heating temperature (*p =* 0.023). t_7*D*_ values differed significantly by heating temperature (*p* = 0.001), as was also the case for shoulder length (*S_L_*) (*p* = <0.0001), where, at lower temperatures, a shoulder was observed versus heating at 93.3 °F. These data aid scientific validation of *Salmonella enterica* inactivation during thermal rendering of poultry blood for use in further processed animal foods.

## 1. Introduction

Rendering of animal carcass components and materials for subsequent conversion into animal and/or human food or feed provides sustainable recycling of resources and a nutritious feed/food stream for consumers, livestock, and companion animals [1]. Nevertheless, raw carcass materials, including blood, present a food safety hazard risk through contamination with microbial human and/or animal pathogens, including *Salmonella enterica* subsp. *enterica*. Multiple studies in recent years have reported the presence of biological food safety hazards such as *Salmonella* on animal carcass components as well as finished rendered products, including blood meal, in the U.S. and in other countries [2,3,4]. Jiang [5] reported that rendered blood meal products contained salmonellae at numbers as high as 240 MPN/g meal. Others reported that *Salmonella* prevalence ranged from 8.7 to 31.3% throughout various rendered products, including poultry meal and blood meal [6,7].

The U.S. Food and Drug Administration Food Safety Modernization Act (FDA-FSMA) and its implementing final rule mandating food safety preventive controls during the manufacture of animal foods (Title 21, U.S. Code of Federal Regulations Section 507) requires covered establishments to scientifically validate process-type preventive controls for identified food safety hazards [8]. Scientific validation of food safety hazard control may be accomplished by various means, including scientific experiments that simulate critical aspects of the antimicrobial process, as well as through other means such as in-plant inoculated product challenge trials that utilize pathogen surrogates exhibiting similar sensitivity to the process as the pathogen of concern [9]. In past research, determination of decimal reduction times (*D*-values) and other thermal inactivation kinetic parameters from testing of pathogen-inoculated products have been developed to assist the validation of effective lethality for commercial food processing. Ceylan and Bautista [10] reported that *S. enterica D*-values ranged from 0.48 to 17.30 min on dried pet food kibble products in a temperature- and moisture content-dependent manner. Bianchini et al. [11] determined that a 5.0 log_10_ CFU/g reduction in *Salmonella enterica* was achieved by heating commercially produced pet food meals to at least 60.6 °C. Our group previously produced *D*-values for multiple serovars of human and avian pathogenic *Salmonella* in poultry offal at 60 °C, reporting the *D*_60__°C_ = 0.89 min for a poultry animal-recovered *S.* Enteritidis [12].

Despite these and other studies reporting the prevalence of *Salmonella* in rendered animal food components or products, or validating thermal lethality to microbial pathogens during rendering, data validating *Salmonella* lethality during rendering of whole blood by for manufacture of blood meal are lacking. Thus, the main objective of this study was to characterize the thermal lethality during simulated commercial rendering of whole chicken blood to human pathogenic *S. enterica*.

## 2. Materials and Methods

### 2.1. Microorganisms and Inoculum Preparation

Isolates belonging to *Salmonella enterica* serovars Senftenberg, Heidelberg, and Typhimurium, recovered from U.S. Department of Agriculture Food Safety and Inspection Service (USDA-FSIS)-inspected chicken harvesting environments (*S.* Typhimurium, *S.* Heidelberg) or procured from American Type Culture Collection (ATCC, Manassas, VA, USA; *S.* Senftenberg 775W ATCC #43845), were chosen from the Food Microbiology Laboratory culture collection (Department of Animal Science, Texas A&M University, College Station, TX, USA). Cultures were revived from −80 °C cryo-storage by inoculating each isolate individually into 10.0 mL sterile brain heart infusion broth (BHIB; Becton, Dickinson and Co., Sparks, MD, USA)—containing tubes and then incubating tubes for 24 h at 35 °C. Following the initial revival, cells from each isolate were aseptically transferred to new, sterile BHI-containing tubes and then incubated in identical fashion (35 °C, 24 h) in order to prepare isolates for subsequent inoculum preparation. Following revival, an inoculum of *Salmonella* isolates (i.e., cocktail) of *S*. Senftenberg 775W, Heidelberg, and Typhimurium was prepared by blending 10.0 mL of each culture into a sterile 50.0 mL conical tube and centrifuging (2191× *g*, 25 ± 2 °C, 15 min) to pelletize cells. After centrifuging the cocktail preparation, the resulting supernatant was discarded and 30.0 mL 0.1% (*w/v*) peptone water (Becton, Dickinson and Co.) was added to wash cells. The resulting suspension of cells was centrifuged again identically, the resulting supernatant was poured off, and the remaining pellet was hydrated with 3.0 mL of 0.1% peptone water. This was completed to yield a 9.0–10.0 log_10_ CFU/mL inoculum in the cocktail.

#### 2.1.1. Preliminary Enumeration of Salmonellae from Non-Inoculated Chicken Blood

In order to verify whether the final inoculum preparation for *S. enterica* was capable of overwhelming any naturally occurring pathogen cells by at least 2 to 3 orders of magnitude, samples of chicken blood were obtained from a cooperating U.S. commercial renderer. Samples were aseptically transported to the Food Microbiology Laboratory in insulated coolers packed with sealed ice packs and serially diluted in 0.1% peptone diluent. Serial dilutions were then inoculated onto Petri dishes containing bismuth sulfite agar (BSA; Becton, Dickinson and Co.) to selectively enumerate salmonellae. Inoculated plates were incubated for 24 to 48 h at 36 ± 1 °C prior to colony counting.

#### 2.1.2. Preliminary Experiment to Verify *Salmonella* Growth for Subsequent Inoculum Preparation

An experiment was conducted to verify that counts of overnight cultures of individual *Salmonella* isolates utilized for the cocktail did not differ from one another, and approximated 9.0 log_10_ CFU/mL, allowing researchers to produce a cocktail with non-differing numbers of each *Salmonella* strain. Revived *Salmonella* strains were aseptically inoculated (10.0 µL each) into sterile 10.0 mL volumes of BHIB and then aerobically incubated without shaking at 36 ± 1 °C for 24 h before enumerating strains individually on tryptic soy agar (TSA; Becton, Dickinson and Co.) poured into Petri dishes. Inoculated Petri plates were incubated for 24 h at 36 ± 1 °C prior to colony enumeration.

#### 2.1.3. Preliminary Experiment to Identify Optimal Medium for Selective Recovery of Non-Injured and Injured Salmonella Cells from Heat-Treated Chicken Blood

An experiment was conducted to identify a chemical supplement to BSA providing effective repair and recovery of sub-lethally injured salmonellae following heating. *Salmonella* cells were revived as described (Section 2.1) and then heated at 93.3 °C for 3.0 min in re-sealable metal vessels (1″ × 6″ galvanized steel by 1″ iron screwcap; SouthLand, Memphis, TN, USA). Immediately after heating, vessels were placed in ice-cold water for 1.0 min to halt further heat-induced inactivation or injury, and samples were extracted thereafter. Surviving *Salmonella* cells were serially diluted in 0.1% (*w/v*) peptone diluent (Becton, Dickinson and Co.) and plated on Petri plates containing TSA (control), BSA without any supplement, BSA ± 0.1% (*w/v*) sodium pyruvate (Sigma-Aldrich Co, St. Louis, MO, USA), BSA ± 0.1% (*w/v*), 3,3′-thiodipropionic acid (TDP; Sigma-Aldrich Co.), or BSA with TSA overlay. Reagents selected were chosen based on the report of Gurtler and Kornacki [13]. Following 24 h incubation at 36 ± 1 °C, counts of *Salmonella*-typical colonies were recorded for determination of the optimal medium for later use in inoculated thermal lethality trials.

### 2.2. Sample Inoculation with Salmonella Cocktail

Raw chicken blood, visually free of carcass tissue, bone, and foreign material, was obtained from the aforementioned commercial rendering establishment (Section 2.1.1). Sample inoculation was achieved by pipetting 0.1 mL of prepared *Salmonella* inoculum into 50.0 mL conical tubes containing 25.0 mL chicken blood and then vortexing for 1 min to homogenize the inoculum in the sample. Following homogenization, serial dilutions of inoculated chicken blood were inoculated on BSA-containing Petri dishes and incubated for 24 h at 36 ± 1 °C to quantify the efficiency of inoculation of microbial cells prior to thermal lethality testing.

### 2.3. Lethality to Salmonella Inoculated into Chicken Blood

Inoculated chicken blood samples were heated to 93.3 °C for 5.0 min to determine total lethality achieved against the pathogen during a simulated commercial rendering process. Inoculation was achieved by pipetting 0.5 mL of *Salmonella* inoculum (Section 2.1) into a 50.0 mL sterile conical tube containing 50 mL chicken blood. Inoculated sample vials were then vortexed for 1.0 min to homogenize the inoculum throughout blood. Samples were then loaded into metal vessels (1″ × 6″ galvanized steel by 1″ iron screwcap; SouthLand) that were then closed and partially submerged in sterile distilled water in a stainless-steel cookpot on an induction-type cooktop. Following heating, vessels were immediately placed in ice-cold water (0 °C) to halt further microbial inactivation. Sample material was aseptically extracted from metal vessels and placed in sterile stomacher pouches, serially diluted with 0.1% peptone diluent, and surviving cells were inoculated on Petri plates. Surviving *Salmonella* were enumerated on Petri plates containing BSA ± 0.1% (*w/v*) sodium pyruvate and then incubated at 36 ± 1 °C for 24 to 48 h prior to colony enumeration.

### 2.4. Temperature-Dependent Thermal Lethality to Salmonella in Chicken Blood

**Figure 1:**
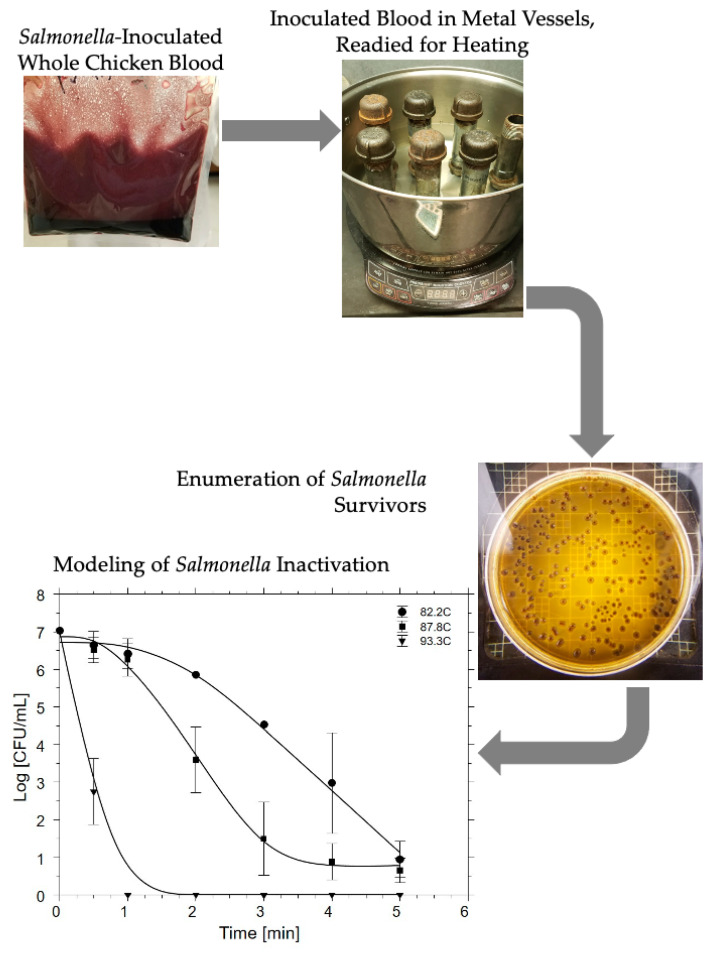
Flow process depiction of *Salmonella* lethality determination from heating of pathogen-inoculated whole chicken blood.

Figure 1 depicts the simple flow of work for the determination of *Salmonella* survival during lethality experiments. Metal vessels (described in Section 2.1.3) were used to help simulate rendering conditions within the commercial rendering establishment with respect to material contacting rendered material during commercial processing. A VWR™ Enviro-Safe^®^ K 50,531 thermometer (VWR Int., Radnor, PA, USA) was set inside an open metallic vessel control filled with 50.0 mL distilled water to monitor temperature readings within sample vessels. Metal vessels were partially submerged in sterile distilled water in a stainless-steel cookpot on an induction cooking device tuned to produce final cook temperatures in the metal vessels of 82.2, 87.8, or 93.3 °C. Vessels were heated to one of the three targeted processing temperatures prior to loading in inoculated blood to pre-heat the vessel but avoid an extended temperature come-up period. This was designed to determine the thermal lethality resulting solely from the simulated commercial rendering process (5 min of heat exposure at one of the three targeted cook temperatures assuming continuous processing in a pre-heated cooking apparatus). Any additional lethality that would be expected to be achieved during commercial rendering come-up periods would thus add to observed lethality.

Once the metal vessel reached the targeted cook temperature, inoculated chicken blood samples were poured immediately into metal vessels, and vessels were sealed with screw-cap closure and replaced in the heated distilled water bath as quickly as possible. One vessel was left unsealed after filling with non-inoculated blood to allow for temperature monitoring. After loading sample vessels into the heating water bath, vessels were removed after 0.0, 0.5, 1.0, 2.0, 3.0, 4.0, or 5.0 min of heat exposure at the experimental temperature. Once removed from heat, metal vessels were immediately placed in ice-cold water (0 °C) to halt further microbial inactivation. Vessels were allowed to cool for at least 1.0 min to ensure complete cooling and allow researcher handling for survivor enumeration. Sample material was thereafter subjected to serial dilution in 0.1% peptone diluent and surviving *Salmonella* were enumerated on BSA supplemented with 1.0 g/L sodium pyruvate to allow recovery and detection of sub-lethally injured salmonellae. Inoculated plates were incubated at 36 ± 1 °C for 24 to 48 h before colony counting. The limit of detection of the assay was 1 CFU/mL (0.0 log_10_ CFU/mL).

### 2.5. Data Analysis

All preliminary and research experiments were replicated three times in identical fashion; experiments for *D*-value determination were designed as a full factorial, randomly assigning samples by three targeted heating temperatures (82.2, 87.8, or 93.3 °C) and seven heating periods (0.0, 0.5, 1.0, 2.0, 3.0, 4.0, and 5.0 min). Plate counts of *Salmonella* were log_10_-transformed prior to data analysis. Survivor data generated from replicates of thermal lethality experiments (Section 2.4) were then fit to non-log-linear models using the freeware Microsoft, Inc. (Redmond, WA, USA) Excel^®^ add-in GInaFiT v.1.7 [14], identifying the best fitting model(s) by use of adjusted R^2^ and root mean square error (RMSE) outputs as indices of a model’s goodness of fit to data. GInaFiT-returned parameters of the fitted model:

(1) $$\log\left(N\right)\;=\;\log\left[10^{\log(N_{o})-\log(N_{res})}e^{-k_{max}t}\left(\frac{e^{k_{max}S_{L}}}{1\;\pm \;\left(e^{k_{max}S_{L}}-1\right)e^{-k_{max}t}}\right)\;\pm \;10^{\log(N_{res})}\right]$$

where *N* is microbial cell count (CFU/mL); *N*_0_ is initial microbial cell count (CFU/mL); *k_max_* is the first order microbial inactivation constant (1/min); *S_L_* is the shoulder length (min), and *N_res_* is the residual microbial cell population (CFU/mL). Additionally, the times required for a 4.0 log_10_-cycle inactivation (t_4*D*_) at each heating temperature were recorded and used for purposes of determining *D*-values and t_7*D*_ times (time required for a 7.0 log_10_-cycle reduction in *Salmonella* counts).

Statistical analysis of microbiological plate counts (log_10_-transformed) calculated *D*-values, t_7*D*_ values, and GInaFiT-returned *S_L_* values were completed by one- or two-way analysis of variance (AOV), as appropriate. Statistically significant differences between least squares means of main effects and/or their interactions (*p* < 0.05) were separated using Dunnett’s test in the case of the preliminary experiment comparing TSA-recovered *Salmonella* counts against other recovery/plating media (Section 2.1.3), or by Fisher’s least squares differences (LSD) test (all others). For all analyses, *p* < 0.05 was set as the significance level. Finally, a *z*-value providing the temperature change required to achieve a 10-fold change in the *D*-value for the process was determined by linear regression analysis of the plot of heating temperatures (*x*-axis) against log_10_-transforms of mean *D*-values (*y*-axis). Data analyses were completed using Prism v9.0 for MacOS (GraphPad Software, LLC, San Diego, CA, USA).

## 3. Results and Discussion

### 3.1. Results of Preliminary Experiments Enumerating Salmonellae from Non-Inoculated Chicken Blood and Overnight Growth of Salmonella Isolates for Inoculum Preparation

Whole chicken blood samples were tested for the presence of contaminating non-typhoidal salmonellae to ascertain the organism’s presence prior to inoculation. The mean number of salmonellae was 4.9 ± 0.6 log_10_ CFU/mL of non-rendered blood; this was significantly higher than previous reports of *Salmonella* numbers on inedible offal, where *Salmonella* loads were reported at 3.7 MPN/100 g [15]. *Salmonella enterica* isolates grew reliably during overnight growth, achieving means of 8.8 ± 0.1, 9.0 ± 0.1, and 9.0 ± 0.1 log_10_ CFU/mL for isolates belonging to serovars *S.* Senftenberg, *S.* Typhimurium, and *S.* Heidelberg, respectively. Results of statistical analysis indicated that mean counts of *Salmonella* isolates did not differ from one another (*p =* 0.069).

### 3.2. Identification of Suitable Medium for Use in Selective Plating and Recovery of Non-Injured and Injured Salmonella Cells from Heated Chicken Blood

**Figure 2:**
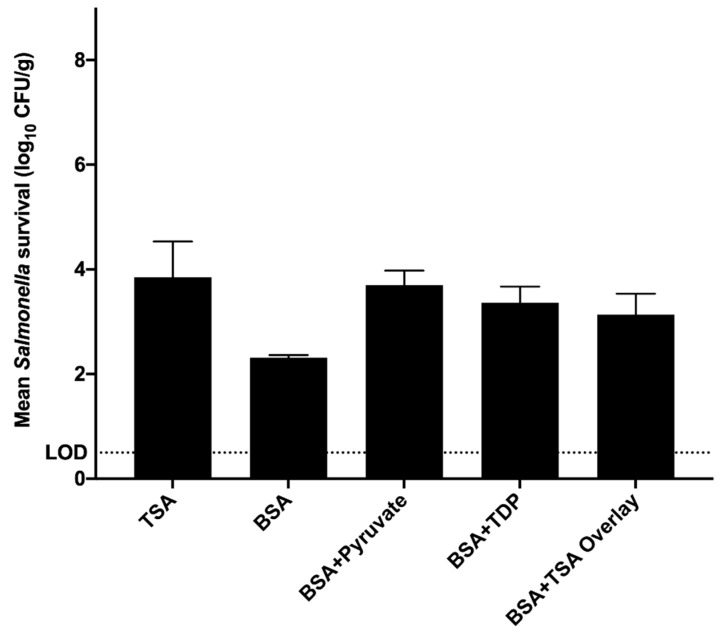
Mean numbers of survivors of *Salmonella enterica* recovered from whole chicken blood subjected to heating for 3.0 min at 93.3 °C (*p* = 0.130; pooled standard error = 0.565). Bars depict means from three identically completed replicates (*N* = 3); error bars present one standard error. *Salmonella* cells were enumerated on tryptic soy agar (TSA), bismuth sulfite agar (BSA), BSA supplemented with 0.1% (*w/v*) sodium pyruvate (BSA ± Pyruvate), BSA supplemented with 0.1% (*w/v*) 3,3′-thiodipropionic acid (BSA ± TDP), or BSA with TSA overlay. *Salmonella* colonies were enumerated after plates were incubated at 36 ± 1 °C for 24–48 h.

Results of preliminary testing to determine which chemical supplement to BSA best facilitated the repair and recovery of injured *S. enterica* inoculated into chicken blood during lethality trials are presented in Figure 2. While statistical analysis indicated no differences between means of recovered *Salmonella* cells based on medium (*p* = 0.130), BSA ± 0.1% pyruvate produced counts of *S. enterica* which numerically differed the least from TSA control counts while also maintaining the selectivity of BSA and was used throughout subsequent experiments.

### 3.3. Total Lethality Achieved in Chicken Blood against Salmonella

Experiments determining the lethality achieved against the *S. enterica* inoculum in chicken blood following 5.0 min continuous heating at 93.3 °C confirmed the ability of commercial rendering to substantially reduce the pathogen, aiding in the manufacture of products not adulterated by *S. enterica*. Numbers of *Salmonella* were reduced to non-detectable counts (detection limit: 0.0 log_10_ CFU/mL) from initial mean counts of 7.4 ± 0.1 log_10_ CFU/mL. Jiang [5] discussed how recent U.S. rendering industry changes have apparently resulted in a decreased prevalence of *Salmonella* presence and transmission than in years past, potentially the result of federal food safety rules requiring industry members to adopt and monitor validated food safety process preventive controls. The U.S. Department of Agriculture Food Safety and Inspection Service (USDA-FSIS) [16] mandates a minimum 7.0 log_10_-cycle lethality for *Salmonella* on human foods derived from poultry, including edible offal. Achieved lethality for *Salmonella* would therefore likely be effective for reducing the pathogen to non-detectable counts in whole blood during rendering, a regulatory requirement for the production of non-adulterated animal food/feeds [17].

### 3.4. Inactivation of S. enterica in Chicken Blood as a Function of Thermal Processing

**Figure 3:**
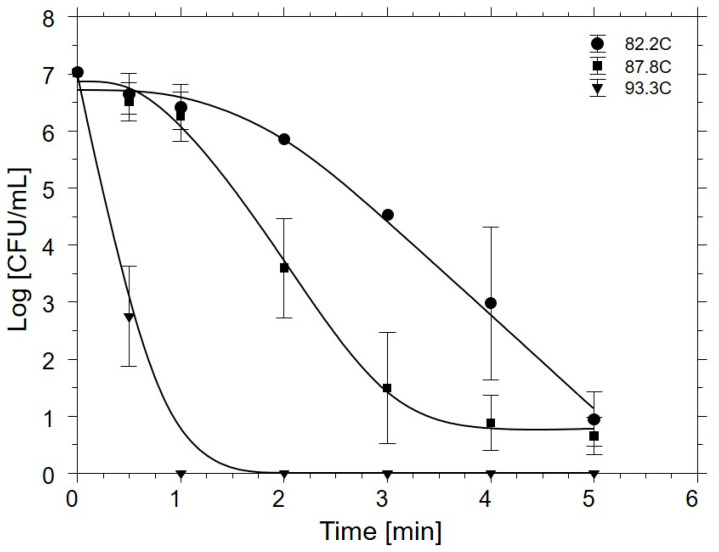
Mean numbers of surviving *Salmonella enterica* in whole chicken blood subjected to heating at 82.2, 87.8, or 93.3 °C. Values depict means from three identically completed replicates (*N* = 3); error bars present one standard error. *Salmonella* cells were enumerated on bismuth sulfite agar supplemented with 0.1% (*w/v*) sodium pyruvate, after plates were incubated at 36 ± 1 °C for 24–48 h.

Figure 3 depicts the inactivation of inoculated *Salmonella* in chicken blood as a function of heating temperature and period. *Salmonella* cells inoculated into blood demonstrated an initial shoulder to survival curve for samples heated at 82.2 and 87.8 °C, with increases in the rates of cell death only observed after approximately 1.0 min of heating. Thereafter, death rates for the pathogen at both temperatures increased, with a characteristic log-linear *Salmonella* count decline for 87.8 °C-heated cells until 3.0 min of heating, after which a tailing effect was observed to occur with a consequent slowing of the death rate. Conversely, 82.2 °C-heated *S. enterica* cells in blood displayed a more rapid inactivation rate after 1.0–1.5 min of heating, presumably due to the *S.* Senftenberg displaying enhanced survival at the lower heating temperature, and from 2.0 min until the 5.0 min mark, the curve was generally log-linear.

Two-way AOV indicated that the interaction of heating temperature by period was highly significant for the survival of *Salmonella* in whole chicken blood (*p* = <0.0001). Counts of the pathogen differed at 0.5 min between samples subjected to the differing heating temperatures, with 82.2 °C- and 87.8 °C-heated cell populations each differing from the count of *Salmonella* cells heated to 93.3 °C (*p* < 0.0001), but not from one another. A similar trend was also observed at 1.0 min of heating, where the counts of 93.3 °C-heated cells (0.0 log_10_ CFU/mL) were significantly lower than either of the populations of *Salmonella* heated to the two lower experimental temperatures (*p* = <0.0001). From 2.0 to 4.0 min of heating, statistical differences became even more pronounced between samples heated to differing target temperatures, where mean populations at each of the three temperatures differed from one another (*p* = 0.043). However, after 5 min of heating, the counts of surviving inoculated salmonellae declined to no more than 0.94 log_10_ CFU/mL, and counts did not differ from one another as a function of heating temperature (*p* = 0.193).

### 3.5. Thermal Death Kinetics for Microorganisms in Chicken Blood as a Function of Heating Temperature

The application of the GInaFiT 1.7 program for the modeling of microbial inactivation allows the user to apply multiple log-linear and non-linear models for the purposes of obtaining predicted inactivation kinetic parameters for a process [14]. In the current study, *D*-values were calculated by re-arrangement of Equation (5) by Valdramidis et al. [18] to:

*D* = (t_4*D*_ − *S_L_*)/4 (2)

using t_4*D*_ and *S_L_* values returned from GInaFiT. Statistical comparison of means of calculated *D*-values and GInaFiT-returned t_4*D*_ values indicated differences occurring between both *D* and t_4*D*_ values as a function of heating temperature (Table 1).

Table 2 reports the model parameters returned by GInaFiT v1.7 for *Salmonella* lethality by heating target temperature. Shoulder lengths for inoculated *Salmonella* cells statistically differed in blood as a function of heating temperature (*p* = <0.0001), with each shoulder length at a specific heating temperature differing from shoulder lengths at the other two temperatures. Observed differences in residual *S_L_* values may have resulted from the inclusion of the high heat-resistant *S*. Senftenberg 775W isolate in the current study and its impacts on apparent inactivation kinetics, especially at the lower heating temperatures. Jones-Ibarra et al. [12] previously reported that the *D*_140__°F_ for *S*. Senftenberg 775W in chicken offal was approximately 6× higher than those for *S*. Enteritidis, *S*. Heidelberg, and *S*. Gallinarum isolates. Vaddella et al. [19] reported *S.* Typhimurium lethality in ground whole chicken carcasses when heated at 78 °C, equivalent to one of the heating temperatures used in the current study. The presence of a shoulder was observed in their data, as was the case in the current study, with accelerated lethality occurring after the initial shoulder. However, multiple log_10_-cycles’ inactivation of the organism required a much longer exposure period for the whole carcass versus that reported here, presumably due to the increased content of fat and bone material providing enhanced thermal insulation as compared to blood. Peleg [20] noted that the existence and duration of a shoulder would be expected to diminish as the intensity of the process intervention, in this heating temperature, was increased, as is the case in the present study.

Goodness of fit parameters (RMSE, adjusted R^2^) both indicated that the fitted models were suitable for the prediction of *Salmonella* lethality during commercial rendering of blood, wherein RMSE declined as heating temperature increased, with a shift to log-linear ± tail being observed at the highest temperature of processing along with a disappearance of the shoulder, and adjusted R^2^ remaining near 1.0 for all experimental heating data. This would indicate that the experimental data did not deviate substantially from model-predicted values, fitting with a high degree of precision (Table 2).

**Figure 4:**
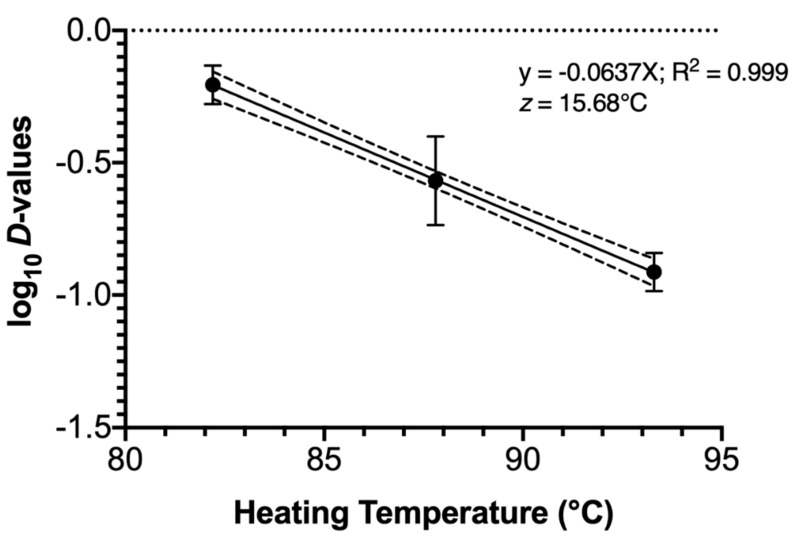
Thermal process constant (*z*-value) for *Salmonella enterica* inoculated and heated in whole chicken blood. Symbols depict mean calculated from three identically completed replicates (*N* = 3); error bars depict one standard error. Dashed lines present the 95% confidence limit boundaries.

The determination of the *z*-value affords the processor the opportunity to alter a process with a predictable outcome in microbial lethality; the *z*-value for the rendering of chicken blood was 15.68 °C (Figure 4). Cautious application of this value is recommended; *z*-values (much like *D*-values) rely on log-linear death kinetics for their determination and do not easily accommodate non-linearity in microbial inactivation. This would not likely be problematic were a commercial renderer desiring to increase the processing temperature in order to increase production throughput but would become increasingly unreliable were the processing temperature decreased, given the presence of non-zero *S_L_* values at lower heating temperatures (Table 2; Figure 3). The *z*-value determined in the current study aligns well with those previously reported (14.1–27.8 °C) against *Escherichia coli* O157:H7 and *Salmonella* in rendered oils [21]. Nevertheless, it is substantially lower than was reported by others testing *Salmonella* lethality in beef-derived carcass materials of 42.8 °C [22]. Nonetheless, those authors reported incorporation of 20% or 50% fat as well as lean meat and connective tissue, much different than the liquid chicken blood free of solid tissue used in the current study.

**Figure 5:**
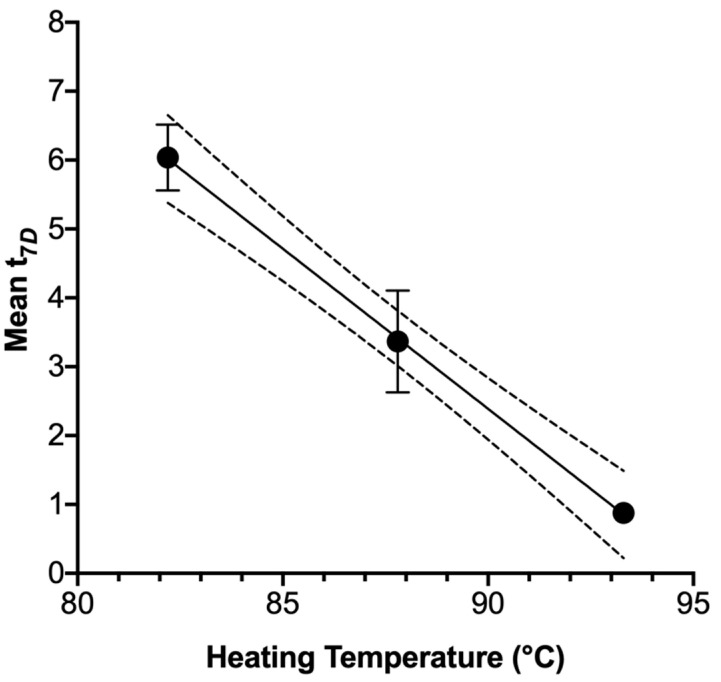
Least squares means of time required to achieve a 7.0 log_10_-cycle inactivation (t_7*D*_) for *Salmonella enterica* heated in whole chicken blood to differing target temperatures. Symbols depict means from three identically completed replicates (*N* = 3); error bars depict one standard error (*p* = 0.001; pooled standard error = 0.73). Dashed lines depict 95% confidence limit boundaries.

Buchanan et al. [23] previously recommended the use of a desired cumulative lethality process, arbitrarily set at t_4*D*_, as compared to designing a process strictly according to a multiple of the *D*-value. Jackson et al. [24] followed this approach for producing a predicted 5.0 log_10_ CFU/g inactivation of *E. coli* O157:H7 cells in ground beef patties during cooking. This method bears a distinct advantage in designing and validating thermal processes when microbial death kinetics are known or suspected to not follow a strictly log-linear kinetic, such as in the current report. The t_x*D*_ metric facilitates the processor accounting for the existence of a shoulder and/or tail in the identification of processing time needed for desired lethality and should produce a more conservative estimate of the needed processing conditions to achieve desired food safety hazard reduction. As was stated above (Section 3.3), the USDA-FSIS [16] mandates a performance objective of a 7.0 log_10_-cycle inactivation of *Salmonella* in poultry-derived human food. In the current study, t_7*D*_ values for *Salmonella* differed by targeted heating temperature, with each t_7*D*_ at a specific heating temperature differing from those of the other two (*p* = 0.001; Figure 5). Juneja et al. [25] previously recommended that food processors apply a t_x*D*_ for achieving a desired lethality performance objective, even recommending the application of the upper limit of a 95% confidence limit for the sake of producing conservative processing schedules for pathogen control. In the present study, mean t_7*D*_ values declined from 6.04 at 82.2 °C, to 3.37 min at 87.8 °C, and to 0.88 min at 93.3 °C. Processing at 82.2 °C or 93.3 °C, each with incorporation of the 95% confidence limit, would require processing periods of 8.09 or 1.45 min at 93.3 °C, respectively (Figure 5). These would represent conservative processes for the rendering of chicken blood, with a high degree of *Salmonella* lethality generally assured.

## 4. Conclusions

The U.S. rendering industry remains in need of scientific validation of the thermal processing systems applied to the manufacturing of components of further processed animal foods and feeds, in order to assist in complying with federal regulations. *Salmonella enterica* was effectively inactivated in whole chicken blood heated to differing endpoint temperatures, yielding *D*-values that differed by heating temperature, as might be expected. Survival curves tended to possess log-linear portions with or without shoulder and/or tail components, overall yielding non-linear survival curves. Time requirements for a 7.0 log_10_ CFU/mL inactivation of *Salmonella* also differed between the three heating temperatures. Finally, a *z*-value of 15.68 °C was produced, giving the temperature change in the process producing a 10-fold change in the *Salmonella D*-value. Data presented demonstrate that the commercial process simulated in this report is effective for achieving significant *Salmonella* inactivation in whole chicken blood for the safe manufacture of blood meal and other derived products not adulterated by the microbial pathogen.

## Figures and Tables

**Table 1:** Least squares means of *D*- (min) and t_4*D*_ values (min) for *Salmonella* inoculated into whole chicken blood and heated to differing target temperatures.

	Heating Temperature (°C)			
	82.2	87.8	93.3	*p* > F; Pooled SE ^2^
*D*-value ^1^	0.61 ± 0.18 ^A^ (0.22, 1.06)	0.41 ± 0.19 ^A,B^ (0.00, 0.88)	0.12 ± 0.02 ^B,C^ (0.04, 0.21)	0.023; 0.13
t_4*D*_	4.12 ± 0.19 ^A^ (3.32, 4.92)	2.42 ± 0.38 ^B^ (0.80, 4.04)	0.50 ± 0.08 ^C^ (0.17, 0.83)	0.0001; 0.35

^1^ Means are determined from three identically completed replicates ± one standard error. Values in parentheses present the lower and upper 95% confidence limits. Means within a row not sharing common letters (^A^, ^B^, ^C^) differ by Fisher’s least squares differences test at *p* = 0.05. ^2^ SE: standard error about mean.

**Table 2:** Least squares means of lethality model parameters for *Salmonella* inoculated into and heated in whole chicken blood.

Model Parameter ^1^	82.2 °C	87.8 °C	93.3 °C	*p* > F; Pooled SE ^2^
*S_L_* (min)	1.62 ± 0.12 ^A^	0.71 ± 0.09 ^B^	0.0 ± 0.0 ^C^	<0.0001; 0.125
*k_max_* (1/min)	3.80 ± 0.29	5.59 ± 0.47	19.71 ± 0.04	
*N_res_* (log CFU/mL)	-- ^3^	0.77 ± 0.17	--	
*N*_0_ (log CFU/mL)	6.71 ± 0.16	6.87 ± 0.19	7.03 ± 0.01	0.089; 0.117
RMSE	0.26	0.23	0.01	
Adjusted R^2^	0.99	0.99	1.00	

^1^ Means are determined from three identically completed replicates ± one standard error. Values not sharing common letters (^A^, ^B^, ^C^) within a row differ by Fisher’s least squares differences test at *p* = 0.05. ^2^ Values depict main effect-specific *p*-value; Pooled SE: standard error about mean. ^3^ -- Indicates no *N_res_* determined from modeling of *Salmonella* survivor data.
